# The War and the Universities

**Published:** 1918-08

**Authors:** 


### The War and the Universities.

The universities of the country are responding grandly to the call of the country for their services. Many of them are furnshing many of their best men to various branches of the service for regular duty, and practically all of their men for such services as they can render in connection with their university work, some of them doing double duty in order that the country may be served, this being done in every case without extra pay.

In research work the universities are taking the lead and are making many scinetific discoveries that are proving invaluable in winning the war. It is especially noticeable that practically all the research efforts of the Committe on Sanitation and Medicine of the Council of National Defense are being conducted by the universities of the country.

In nearly every state the universities are spending their energies and their appropriations to train men and women for war work. At the University of Texas over 5,000 students are now taking courses in aviation, radio operation, auto mechanics and other thing that will fit them especially for serving the country, and thousands have alerady gone from this institution into active servise.

But for the universities the country would have been poorly prepared for the war.

